# Special issue on Six Sigma metrics - experiences and recommendations

**DOI:** 10.11613/BM.2018.020301

**Published:** 2018-06-15

**Authors:** Sten Westgard, Hassan Bayat, James O Westgard

**Affiliations:** 1 Westgard QC, Madison, USA; 2 Immunogenetics Research Center, Mazandaran University of Medical Sciences, Sari, Iran

**Keywords:** Six Sigma, Sigma metrics, quality control

In the dawn of the laboratory medicine era, we had a different view of quality. Back when we were pipetting tests by mouth, and we were constantly tinkering with the instrumentation, and the patient volumes were, by today’s standards, miniscule, we didn’t mind as much error. Error rates of 1%, 5%, 10%, that wasn’t so bad.

In the last half century, so much has changed, not only in the laboratory but in the expectations of quality management. What was once acceptable in terms of defect rates has now become unsustainable for today’s automated high volume operations. When many laboratories are routinely reaching millions of reportables *per* year (some even *per* month!), even a 1% error rate is ruinous to the laboratory’s ability to produce reliable turn-around times and accurate results.

All around us, the laboratory has made a quantum leap forward in output and effectiveness. Instruments have been engineered to better precision, as automation has eliminated many error-prone processes, and as informatics has made communication of massive numbers of results possible. Without all of these improvements, laboratories would not have been able to keep up with the clinical demand.

But our expectation of quality has not evolved commensurately. Many laboratories retain the old mindset of 1%, 5%, 10% error rates as acceptable. They focus on pre-analytical and post-analytical processes, leaving the analytical quality of their tests unexamined, unverified, and unimproved. Laboratories that ignore their analytical quality do so at their own peril, generating the wrong results faster and more cheaply than ever. As they transform laboratories into number factories, they threaten to turn themselves into mere commodities, interchangeable cogs. The future for a laboratory that doesn’t focus on quality is not only consolidation, but possibly extinction itself. If we don’t value quality, don’t prove quality, don’t rigorously insist on quality in our tests, we are the just the latest cobblers, saddle-markers, type-writer repairmen, and apothecaries to see our profession erased by technological change.

Six Sigma and analytical Sigma metrics provide a solution to our challenges. Not only through Six Sigma can we identify when our methods are actually appropriate for clinical care, it can help change how much quality control (QC) we have to do, guide our risk management efforts, even make changes to the operational expenditures. This special issue of *Biochemia Medica* is a small sampling of how analytical Sigma metrics are being applied around the world.

Six Sigma has not been in the laboratory for very long – the first study that benchmarked laboratory quality on the Six Sigma scale came out in 2001, by David Nevelainen (*1*). So with less than two decades of application, it is a relatively new field, one that is not without its controversy, challenges and debates. But since 2001, a suite of tools have been developed to allow laboratories to harness the power of Six Sigma to assess method quality, optimize QC procedures, change the number of rules and number of controls being run, and most recently, even change the frequency of QC. Virtually all the questions we can ask about how and when to run QC can now be answered by the analytical Sigma metrics, and we provide a review of these techniques and developments in one of the cornerstone articles in this issue. Xuehui Mao *et al.* show the application of Sigma metrics to assessing the quality of an instrument in a laboratory, the basic implementation that every laboratory can consider (*2*). Yong Xia *et al.* take the application one step further, incorporating Sigma metrics into the traditional risk assessments that connect test results to patient care (*3*). Cao and Qin show how analytical Sigma metrics can be used to evaluate whether or not third-party reagents are fit for purpose, a key question in much of the developing world – where there is strong temptation to use a cheaper local reagent rather than the original manufacturer’s reagent; the assumption that reagents are interchangeable is too often unexamined (*4*). At the other end of the testing lifecycle, Petrides and Schneider show how diagnostic manufacturers are incorporating Sigma metrics into the earliest stages of assay design (*5*).

Analytical Sigma metrics did not emerge out of a vacuum, they were built on a foundation of work completed over previous decades in critical systematic error, allowable analytical total error, and even the earliest equations that combined imprecision and bias into the global standard of analytical total error (*6*). There are debates and controversies that have raged for decades about these foundations and mathematical models (*7*-*9*). Hassan Bayat provides the most technical paper in this issue, delving into the details of the analytical Sigma metric to reveal what we can calculate and what we may actually observe (*10*). Wyzte Oosterhuis details an alternate Sigma metric approach that avoids possible flaws in total allowable errors built from data on biological variation; even as we see our analytical goals tighten, Oosterhuis and Coskun note that we may still be allowing "too much error" to exist in our methods (*11*). They believe that while we are heading down the right road, we’re using the wrong map (model). It may be that both our maps are practically the same, and the differences are only of interest to the seasoned cartographer, not the casual traveler.

Another of the largest and most obvious challenges of Six Sigma is the selection of the appropriate performance specification. How good does a test need to be? As anyone who looks are Clinical Laboratory Improvement Amendments (CLIA), The College of American Pathologists (CAP), Reference Institute for Bioanalytics (Rilibak), and The Royal College of Pathologists of Australasia external quality assessment goals can tell you, the world has not reached a consensus. In Germany, you may have a very large interlaboratory comparison goal for calcium, while those labs in Australia have a much tighter goal, and those trying to achieve a biologically derived goal might find the challenge still harder. There is no standardization of allowable total error performance specifications for laboratory tests around the world. As long as external quality assurance (EQA) programs are competing against each other for business, it’s unlikely that they will harmonize or standardize (wider goals provide a financial advantage, making for happier customers). If we can’t agree on a standard performance specification, it makes our calculated analytical Sigma metrics incomparable. Guo *et al.* show the differences in Sigma metrics that can be calculated when Chinese national performance specifications are used *vs* the United States CLIA/CAP goals (*12*). We showed how a standardization protocol in Egypt is being introduced to encourage all laboratories to use the same quality goals for their Sigma metrics benchmarks (*13*). Finally, Varela and Pacheco introduce a new evaluation matrix that allows laboratories to assess which performance specification may be most appropriate for use (*14*).

The contribution to the advancement of QC practices and efficient operation of the laboratory through Six Sigma cannot be ignored. Laboratories, even as they acknowledge the debates and challenges that will continue to feature in the use of Six Sigma, need to begin implementing Sigma metrics to optimize their practices. Future demands to “do more with less” – our constant mantra – can only be realized through adoption of advanced quality techniques such as Six Sigma.
